# Executive Search Consultants’ Biases Against Women (or Men?)

**DOI:** 10.3389/fpsyg.2020.541766

**Published:** 2020-11-03

**Authors:** Rudolf Siegel, Cornelius J. König, Yannik Zobel

**Affiliations:** Work and Organizational Psychology, Saarland University, Saarbrücken, Germany

**Keywords:** headhunting, executive search, sex discrimination, own-gender bias, implicit measure

## Abstract

Women remain under-represented in leadership positions in many countries. Since executive search consultants (also known as headhunters) act as gatekeepers in the hiring process, headhunters’ biases might influence the female under-representation. There is preliminary evidence that suggests headhunters favor men, but direct evidence is missing. Thus, this study directly tested this assumption using implicit and explicit measures (an implicit association test and a gender role attitudes survey), completed by 123 German executive search consultants. Although neither measure showed an anti-women bias (with the explicit measure being compared to a match sample from a representative survey using propensity score matching), the implicit association test showed an in-group bias (i.e., male headhunter had a stronger association of men and competence than of women and competence). The latter is worrisome because the majority of consultants in this business are men. Thus, organizations interested in more female managers need to carefully consider who they hire as their executive search consultants.

## Introduction

Women remain under-represented in top leadership positions – for example, only 5.0% of CEO positions and 26.5% of senior-level positions in S&P 500 companies in the U.S. are currently occupied by women (Catalyst, 2019). This situation is similar in many other countries (Aluchna and Aras, 2018). For example, German women remain under-represented in leadership positions, not only in senior leadership positions (where 26% of the positions are held by women) but also on lower management levels (where 40% of the positions are held by women; Kohaut and Möller, 2019).

Women’s under-representation in leadership is often in contrast with the societal norms. If we take the example of Germany, representative data from the German population shows a trend toward the endorsement of gender-egalitarian statements (Baier, 2014). Although this trend might have come to a standstill before reaching gender equality, a considerable number of Germans, men and women, seem to support the goal of gender equality (Lois, 2020).

Several societies have responded to this discrepancy between gender norms and gender differences in leadership positions by changing the law, for instance, within the European Union (see Leszczyńska, 2018). We can again use Germany as an example where a new statutory from 2016 requires a 30% share of women in supervisory boards (Holst and Kirsch, 2016). However, only organizations that must oblige to this 30% share of women in supervisory boards reach this figure, whereas executive boards remain rather unchanged and dominated by men (Holst and Wrohlich, 2017), suggesting that such a law results only in minimal changes beyond the target group (i.e., beyond supervisory boards). This indicates the necessity to look beyond legal changes if women’s under-representation in leadership position is to be changed.

One group of people who influence who gets leadership positions are executive search consultants, also known as headhunters (Khurana, 2002; Faulconbridge et al., 2009; Hamori, 2010). It is their job to identify possible candidates, evaluate them, and present a shortlist to decision makers in organizations (with a shortlist consisting of people considered suitable for the vacant position; Finlay and Coverdill, 2002). Executive searches have become a large business, with estimated global revenues of members of the field’s professional association being 14 billion US dollars in 2017 and rising (Association of Executive Search and Leadership Consultants, 2018). In Germany, revenues of executive search firms were rising steadily up to almost 2.5 billion Euros in 2018 and an expected growth of 6.7% for 2019 (Bundesverband Deutscher Unternehmensberater, 2019).

Given the important gatekeeping role of executive search consultants in the hiring process of managers, there could be major implications if these consultants hold the same stereotypes against females as the general population. Until today, gender stereotypes still exist in the general population: Women are seen as more communal, and particularly men characterize women as less agentic than men (Hentschel et al., 2019; Eagly et al., 2020). Furthermore, prejudices against women in leadership positions are still common (e.g., as shown in a German sample using an indirect interview technique that controls for socially desirable responding; Hoffmann and Musch, 2019). If headhunters believe that women do not have the right attributes to be successful top managers, they will likely prefer male candidates, thus putting women at a disadvantage. Stereotypes could be particularly relevant in the executive search industry because this industry has been described as one in which there needs to be the “right chemistry” between applicants and organizations (Finlay and Coverdill, 2002; Steuer et al., 2015).

Preliminary evidence is consistent with the idea that gender matters for executive search consultants. In particular, Tienari et al. (2013) conducted qualitative interviews with executive search consultants and found that exclusion of women can happen at each step of the process (identifying and profiling candidates, shortlisting, and presenting candidates to organizations). For example, one consultant describes that he is only willing to promote a female candidate if he has “a good feeling” about her, which he does not require for a male candidate (p. 54). Furthermore, Dreher et al. (2011) findings suggest that executive search consultants identify White males as potential candidates more often than people from other groups. This can be explained by role congruity theory: The better stereotypes of job requirements and one gender match, the more likely it is that this gender is positively evaluated (Koch et al., 2015).

Despite the importance of headhunters’ biases against women, research has not directly assessed this bias, and the aim of this study was therefore to do this by using an implicit (and an explicit) measure in a German sample. Implicit measures have been developed to assess for automatic and subtle, potentially unintentional processes that influence behavior such as stereotypes (e.g., Kurdi et al., 2019). This study uses the most common implicit measure, the implicit association test (IAT; Greenwald et al., 2003). In the IAT, participants have to categorize stimuli together in varying pairs, so that the time they need to complete the categorizations reflects strength of the underlying associations (in our context between women and competence vs. between men and competence). We hypothesize that headhunters have a stronger association of men and competence than of women and competence in this IAT.

The IAT is complemented by an explicit measure in which headhunter are directly asked for their attitudes (Walter, 2018). Given the problem of social desirability when responding to sensitive topics (e.g., Krumpal, 2013), we refrain from expecting that headhunters have a bias against women in this explicit measure.

## Materials and Methods

The hypotheses, variables, and analyses were preregistered before conducting this study.^1^ The preregistration included an additional measure for ethical behavior, but the analysis of this variable is not part of this paper (but can be made available on request).

### Participants

We used search engines like Google and headhunting websites (e.g., www.headhunterindeutschland.de) to collect mail addresses of German executive search consultants. We personally invited 2,006 consultants *via* email [644 females (32%) and 1,362 males (68%)] to participate in an online study, programmed with the survey software SoSci Survey (Leiner, 2016). Of these, 204 started the study and 139 completed it. The final sample size consisted of 123 consultants because of the exclusion of 11 participants (due to technical problems); three participants were also excluded because they stated they had work experience outside the interval of 2–50 years (i.e., either not enough experience or unrealistically long experience) and two participants who had too many invalid answers in the IAT more than 10%, the threshold suggested by Greenwald et al. (2003).

Of these, 48 were females (39%) and 75 males (61%). On average, participants were 49.9 years old (*SD*=10.6) with an overall work experience of 26.1 years (*SD*=11.2) and an average of 12.3 years (*SD*=6.1) experience working as an executive search consultant. They typically worked for several industries (number of industries *M*=4.4, *SD*=11.2), with the most common industries being engineering (55%), vehicle manufacturing (46%), and information technology (40%). Female and male headhunters did not differ in the sectors they worked for [all $\chi^{2}\left(1\right)\leq 4.16$, ps≥0.786, ps adjusted for multiple testing]. On average, they reported working for 15.9 organizations (*SD*=12.4) that hired them to search for executive positions. Participants reported to accept on average 29.7 search assignments (*SD*=29.64) and to shortlist on average 7.3 candidates per assignment (*SD*=9.8, *Mdn*=5).

### Measures

#### Implicit Bias Measure

To measure participants’ implicit biases against women, we used SoSci Survey’s implicit association test module because it follows the recommendations of Greenwald et al. (2003). This module allows web IAT testing using any kind of stimuli,^2^ and we relied on the gender-competence IAT that was developed by Ebert et al. (2014 Study 1b). In this IAT, participants are asked to categorize the targets “man,” “male,” “woman,” and “female” as well as the attributes “competent,” “capable,” “incompetent,” and “incapable” (all stimuli were presented in German to participants). The stimuli were sequentially presented on a screen. Once a stimulus appears, participants have to categorize the targets and attributes. A target and an attribute share the same keyboard key for categorization. The assumption is that people react more quickly to target-attribute combinations that are congruent with people’s associations than to combinations that are incongruent. For instance, if someone associates females with competence, reaction times should be lower in cases where the stimuli “woman,” “female,” “competent,” or “capable” share the same key compared to cases where the stimuli “man,” “male,” “competent,” or “capable” share the same key.

IATs within SoSci Survey, following Greenwald et al. (2003), consist of seven blocks: five practice blocks and two test blocks. A practice block consists of 20 trials and a test block of 40 trials. The blocks are separated by a participant-paced break where a short description of the task is displayed. Within each block, stimuli are presented in random order. A stimulus stays on screen until participants press the correct associated key. If participants make an error, a red cross appears until participants press the correct key. A new stimulus appears after a pause of 250ms after each correct response. The target and attribute concepts are shown in the upper corners throughout the experiment. The location of the word to be categorized (e.g., “man” etc., in our IAT) is always in the middle of the screen. The IAT version for mobile users (used by n=8) shows two buttons for responding as opposed to key pressing as an input. There were no differences in IAT D effect between the desktop and mobile version (tested with a bootstrapped Welch-*t*-test to take differing group sizes into account, *t* = 1.81, *p* = 0.098, with 5,000 samples), and we did therefore not include it as a covariate in the analyses.

In the first practice block of our IAT, the instruction asked half of the participants to hit the letter “e” when they see “man” or “male” on the screen and the letter “i” when they see “woman” or “female.” (For the other half, the letters were reversed. Thus, the experiment was counterbalanced.) In the second practice block, the instruction asked these participants to hit the letter “e” when they see “competent” or “capable” on the screen and the letter “i” when they see “incompetent” or “incapable.” In the third practice block, participants’ tasks were to categorize all eight words (i.e., hit “e” when seeing either “man,” “male,” “competent,” or “capable” and hit “i” when seeing either “woman,” “female,” “incompetent,” or “incapable”). The fourth block was the same as the third practice block but was a test block. The fifth block was similar to the first but the correct responses (i.e., the “e” and “i”) were reversed. In the sixth (practice) block, participants were again asked to categorize all eight words but with reversed responses (i.e., hit “e” when seeing when seeing either “woman,” “female,” “incompetent,” or “incapable” and hit “i” either “man,” “male,” “competent,” or “capable”). The seventh block was again a test block.

As suggested by Greenwald et al. (2003), an improved D score was calculated as a measure of bias. A D value greater than zero reflects a stronger association between men and competence than between women and competence. To calculate the D score, the mean of trial latencies in the incongruent test block is subtracted from the mean of trial latencies in the congruent block. This raw difference score is then divided by the standard deviation of all trial latencies to obtain a standardized measure (i.e., the D score). We used the procedure suggested by Kurdi et al. (2019) to calculate the internal consistency (i.e., split-half reliability), adapted to the improved D score and using 1,000 iterations, and found a mean value for our IAT of $r_{M}=0.75$ (*SD*=0.03).

#### Explicit Bias Measure (and Comparison Group)

To measure participants’ explicit gender attitudes, we used the Gender Role Attitudes Scale that was developed for the German General Social Survey “ALLBUS 2016” (Wasmer and Baumann, 2018), part of a time series of cross-sectional surveys with representative samples. This gender role attitude measure consists of nine items (for the German original see GESIS – Leibniz-Institut für Sozialwissenschaften, 2017; for the development of the scale see Walter, 2018; for an English translation see Wasmer and Baumann, 2018). Sample items are: “A full-time working mother can normally establish just as close a relationship with her small child as a mother who does not work” and “The best way to organize family and work life is for both partners to work full-time and to look after the home and children equally.” Respondents answered on a scale from 1 = completely agree, 2 = tend to agree, 3 = tend to disagree, to 4 = completely disagree. Higher scores mean agreement of traditional gender role attitudes.

In the ALLBUS 2016 survey, which is freely available (GESIS – Leibniz-Institut für Sozialwissenschaften, 2017), 1,740 participants answered these items, resulting in a Cronbach’s alpha of 0.79 for this scale. In our sample of executive search consultants, we also achieved a Cronbach’s alpha of 0.79

Because it is difficult to argue that there is a meaningful threshold that indicates modern vs. traditional gender role attitudes, we compared our sample of headhunters to a matched sample from the population assessed by the ALLBUS 2016 survey. To select the best control match for every participant in our headhunter sample, a propensity score matching was used (following the recommendation of West et al., 2014) and using the MatchIt R package (Ho et al., 2011). A logistic regression model was chosen to estimate a propensity score that is defined as the probability of being in the headhunter sample based on covariates. We used age, gender, and education as covariates and fixed gender and educational level to be exactly the same for an individual in the headhunter sample and the matching control (see Table 1 for a summary of the samples before and after matching). Participants who indicated “apprenticeship” as education level (n=3) were excluded from matching because this education level could not be matched to the ALLBUS sample. Thus, matching resulted in a sample of N=120 per group. To examine the similarity of the headhunter and control group, the standardized bias was used, with a standardized bias of a certain covariate that is less than 0.25 (absolute value) being evidence for good matching (Ho et al., 2007). It is defined as the weighted difference in means divided by the standard deviation of the original full comparison group (Ho et al., 2007). All covariates were below this threshold. The only exception (education level “middle school,” *M*=–6.40) seems ignorable because only one person belonged to this education level.

**Table 1:** Means, standard deviations, absolute, and relative counts of covariates used for the propensity score matching procedure.

	Headhunter sample		ALLBUS sample	
	Before matching *N* =123	After matching *N* =120	Before matching *N* =1740	After matching *N* =120
Age	49.4 (10.4)	49.4 (10.5)	50.8 (17.8)	49.3 (10.5)
Gender (female)	48 (39.0%)	46 (38.3%)	858 (49.3%)	46 (38.3%)
Education:				
Higher education entrance qualification	119 (99.2%)	119 (99.2%)	686 (39.9%)	119 (99.2%)
Intermediate school-leaving certificate	1 (0.8%)	1 (0.8%)	1,019 (59.3%)	1 (0.8%)
No school-leaving certificate	0 (0.0%)	0 (0.0%)	14 (0.8%)	0 (0.0%)

For age, means and standard deviations (in parentheses) are given. For categorical variables (sex and education) absolute and relative (in parentheses) frequencies are given. ALLBUS sample = the comparison sample from the German General Social Survey “ALLBUS 2016” (Wasmer and Baumann, 2018).

## Results

Table 2 gives an overview of descriptives and correlations for the measured variables. To test for differences in the explicit gender attitude measure we conducted a Welch’s *t*-test: headhunters (*M*=1.84, *SD*=0.53) and matched ALLBUS control sample (*M*=1.87, *SD*=0.57) did not differ, $t\left(237.12\right)=0.48,p=0.631,d=0.06$, as expected.

**Table 2:** Means, standard deviations, and intercorrelations of study variables.

		*M*	*SD*	1	2	3	4
1	Gender role attitudes	1.84	0.54	(0.79)	0.21^*^	0.08	−0.26^**^
2	IAT effect	0.00	0.43		(0.75)	0.25^**^	−0.59^***^
3	IAT order	1.51	0.50			–	−0.05
4	Gender	1.39	0.49				–

N =123. IAT = implicit association test measuring gender bias (with higher scores indicating an implicit pro-men bias). Higher gender role attitudes indicate an explicit pro-men bias. Reliability estimates in brackets. Coding of IAT order: 0 = male/competence first, 1 = female/competence first. Coding of gender: 1 = male, 2 = female.

* p < 0.05;

** p < 0.01

*** p < 0.001.

Analyzing the implicit measure, we first tested for differences between IAT order (male and competence paired in the first test block vs. female and competence paired in the first test block). Since we found a significant difference, $t\left(112.82\right)=-2.85,p=0.005,d=0.52$, the IAT overall effect was calculated using a regression that controlled for IAT order (see Table 3). In this table, the IAT overall effect is reflected by the intercept, $b_{0}=-0.11$, 95% CI $\left[-0.22,-0.01\right]$, p=0.039. The value of the intercept indicates overall a stronger association between women and competence than men and competence.

**Table 3:** Results of regression analyses predicting the implicit association test (IAT) effect (*N* = 123).

	IAT effect			
	Step 1		Step 2	
	*b*	95% CI of *b*	*b*	95% CI of *b*
Intercept	−0.11^*^	[-0.22, -0.01]	0.10	[0.00, 0.20]
IAT order	0.22^**^	[0.07, 0.37]	0.19^**^	[0.07, 0.31]
Gender			−0.51^***^	[-0.63, -0.39]
$R_{adj}^{2}$	0.06^**^		0.39^**^	
$\Delta R_{adj}^{2}$			0.33^***^	

An intercept greater than zero reflects a stronger association between men and competence than between women and competence. Coding of IAT order: 0 = male/competence first, 1 = female/competence first. Coding of gender: 0 = male, 1 = female.

* p < 0.05;

** p < 0.01;

*** p < 0.001.

For exploratory purposes, we also took gender into account, and the regression model improved significantly, $\Delta F\left(1\right)=67.25,p<0.001,\;\;\Delta R_{adj}^{2}=0.33$ (see also Table 3). The effect of gender was b=−0.51, 95% CI $\left[-0.63,-0.39\right]$, p<0.001, meaning that female participants had a lower IAT effect than men. Separate regression analyses for both gender (controlling for IAT order, see Table 4) indicates that female headhunters implicitly associated women stronger with competence, and male headhunters associated men stronger with competence, which implies an implicit in-group bias for both genders. A similar pattern was also observable in the explicit measure, indicating more traditional gender roles for male (*M*=1.96, *SD*=0.54) than for female (*M*=1.67, *SD*=0.48) headhunters, $t\left(109.15\right)=3.07$, p=0.003, d=0.59, 95% CI $\left[0.20,0.97\right]$.

**Table 4:** Results of regression analyses predicting the implicit association test (IAT) effect separately for male (*n* = 75) and female (*n* = 48) headhunters.

	IAT effect			
	Male headhunters		Female headhunters	
	*b*	95% CI of *b*	*b*	95% CI of *b*
Intercept	0.12^*^	[0.01, 0.23]	−0.44^***^	[-0.58, -0.29]
IAT order	0.16^**^	[0.01, 0.31]	0.24^*^	[0.04, 0.44]
$R_{adj}^{2}$	0.04^*^		0.09^*^	

An intercept greater than zero reflects a stronger association between men and competence than between women and competence; an intercept lower than zero reflects a stronger association between women and competence than between men and competence. Coding of IAT order: 0 = male/competence first, 1 = female/competence first.

* p < 0.05;

** p < 0.01;

*** p < 0.001.

## Discussion

This study aimed to explore whether executive search consultants have implicit or explicit biases against women. Compared to representative data regarding an explicit measure of gender bias, search consultants did not show a relevant mean difference. We also used the IAT to test for implicit bias, and although we did not find a general bias against women, our data revealed an in-group bias toward the headhunter’s own gender: Male headhunters had a stronger implicit association of men with competence, whereas female headhunters had a stronger implicit association of women with competence. A similar trend was also found in the explicit measure, where male headhunters endorsed more traditional gender roles than female headhunters.

The results regarding in-group (or own-gender) biases are in line with previous research with implicit measures (e.g., Ebert et al., 2014; Leach et al., 2017) and with explicit measures (e.g., Lois, 2020). However, within the given circumstances a pro-men bias among male search consultants is worrisome because the majority of consultants seems to be men. Not only is this the case for our sample where 61% of the actual respondents (and 68% of the invited participants) were male, but also among the consultants in the US, where 73% of the most influential headhunters (McCool, 2008; see also Dreher et al., 2011) and 86% of the primary contact people of the top 50 recruiting companies are male (Hunt Scanlon top 50 recruiters, 2018). In other words, if the majority of executive search consultants implicitly (or explicitly) believe that men are more competent than women, they might not try hard enough to identify, profile, and shortlist female candidates, and this might contribute to the dominance of men in top management positions.

It should be kept in mind that the non-significant difference between our sample of executive search consultants and a representative German sample in the explicit measure does not indicate that our sample does not hold any explicit stereotypes for two reasons. First, this is only a test of a mean difference, and values always deviate around the mean. Second, we found a mean of around 1.8 (i.e., closer to “tend to disagree” than to “completely disagree” with traditional gender roles), and whether such a value is evidence for an explicit stereotype against women is open to personal interpretation.

A noteworthy limitation is that this study focused only on gender and did not include biases based on other social identities (e.g., race) and characteristics (e.g., weight). In particular, researchers have suggested that executive search consultants also have a racial bias toward white people (Dreher et al., 2011; Holgersson et al., 2016). Future research should therefore use implicit and explicit test for racial biases as well as biases against other groups. Furthermore, a discussion of a study using the IAT would be incomplete without mentioning that there is a considerable controversy around the IAT as a measure of implicit bias (e.g., Mitchell and Tetlock, 2017; Payne et al., 2017; Jost, 2019), and we thus welcome replications using other (implicit) measures.

The results have important implications for practitioners: If organizations are interested in increased gender diversity of their management, they should carefully consider who they want to hire as their executive search consultant. Furthermore, professional organizations such as the Association of Executive Search and Leadership Consultants need to continue their educational efforts so that people in the industry are aware of subtle biases that can distort their search process and should provide trainings that can reduce the impact of biases in the search process. For example, Devine et al. (2012) developed a training that reduced implicit stereotypes by replacing stereotypical responses, taking the perspective of the minority, and imaging counterstereotypic others. Such training might also be beneficial in the executive search consultancy context.

## Data Availability Statement

All data and analysis files necessary to reproduce our findings are available at https://osf.io/tpy96/.

## Ethics Statement

Ethical review and approval was not required for the study on human participants in accordance with the local legislation and institutional requirements. The patients/participants provided their written informed consent to participate in this study.

## Author Contributions

RS, CK, and YZ: designed the study and interpreted the results. YZ: collected the data. RS and YZ: analyzed the data. RS and CK: wrote the paper. All authors contributed to the article and approved the submitted version.

### Conflict of Interest

The authors declare that the research was conducted in the absence of any commercial or financial relationships that could be construed as a potential conflict of interest.

## Footnotes

^1^https://aspredicted.org/sq2su.pdf

^2^https://www.soscisurvey.de/help/doku.php/en:create:questions:iat
